# The Association Between Neuropathic Pain, Pain Intensity, and Inflammatory Activity in Rheumatoid Arthritis

**DOI:** 10.3390/jcm15041601

**Published:** 2026-02-19

**Authors:** Zeynel Abidin Akar, Dilan Yıldırım, Ömer Karakoyun, Kadir Kaya, Mehmet Çağlayan, Pelin Oktayoğlu, Remzi Çevik

**Affiliations:** 1 Division of Rheumatology, Department of Physical Therapy and Rehabilitation, Faculty of Medicine, Dicle University, 21300 Diyarbakır, Türkiye; dilanyildirim484@gmail.com (D.Y.); ftrmehmet63@hotmail.com (M.Ç.); plnftr@hotmail.com (P.O.); 2 Department of Dermatology and Venereology, Faculty of Medicine, Dicle University, 21280 Diyarbakır, Türkiye; omerkarakoyun@gmail.com; 3 Department of Dermatology and Venereology, Faculty of Medicine, İstinye University, 34010 İstanbul, Türkiye; drkartalkaya@hotmail.com

**Keywords:** rheumatoid arthritis, neuropathic pain, PainDETECT, pain intensity, quality of life, disease activity

## Abstract

**Background**: Nociplastic-like pain features are increasingly recognized as significant contributors to chronic pain and reduced quality of life in patients with rheumatoid arthritis (RA). However, their clinical correlates and relationship with disease activity remain incompletely understood. **Objective**: To evaluate the prevalence of nociplastic-like pain features in patients with RA and to investigate their associations with disease activity, pain intensity, fatigue, sleep quality, and health-related quality of life. **Methods**: In this cross-sectional study, 160 patients with RA were enrolled. Nociplastic-like pain features were assessed using the PainDETECT questionnaire. Disease activity was evaluated using the Disease Activity Score in 28 joints (DAS28). Pain intensity, fatigue, sleep quality, and health-related quality of life were assessed using the visual analog scale (VAS), Functional Assessment of Chronic Illness Therapy–Fatigue (FACIT-F), Pittsburgh Sleep Quality Index (PSQI), and Short Form-36 (SF-36), respectively. Continuous variables were compared using *t*-tests or Mann–Whitney U tests according to data distribution. Stepwise multivariate linear regression analysis was performed to identify independent factors associated with PainDETECT scores. **Results**: Pain patterns suggestive of nociplastic-like features were identified in 22.5% of patients. These patients had significantly higher pain intensity, greater fatigue (lower FACIT-F scores), poorer sleep quality (higher PSQI scores), and lower SF-36 scores across all domains compared with patients without these features (all *p* < 0.001). PainDETECT scores showed a strong positive correlation with VAS pain intensity (r = 0.679, *p* < 0.001) and a moderate correlation with DAS28 (r = 0.536, *p* < 0.001). PainDETECT scores were negatively correlated with FACIT-F (r = −0.512, *p* < 0.001) and several SF-36 domains. In stepwise multivariate regression analysis, pain intensity, tender joint count, and education level emerged as independent predictors of nociplastic-like pain features, whereas inflammatory markers (CRP, ESR) and DAS28 were excluded from the model. **Conclusions**: Nociplastic-like pain features are common in RA and are independently associated with pain intensity, joint tenderness, and psychosocial factors rather than inflammatory activity alone. Routine assessment of these features is essential for personalized pain management and underscores the importance of considering potential central sensitization mechanisms in addition to traditional anti-inflammatory therapies.

## 1. Introduction

Rheumatoid arthritis (RA) is a chronic systemic autoimmune disease characterized by persistent synovial inflammation, leading to progressive joint damage, functional disability, and reduced quality of life [1,2]. Pain is among the most common and disabling symptoms in RA and profoundly affects physical functioning, psychological well-being, and social participation [1,3]. Traditionally, pain in RA has been attributed primarily to synovial inflammation; however, growing evidence indicates that RA-related pain arises from complex, multimodal mechanisms, involving both nociceptive and nociplastic (non-nociceptive) processes [2,4,5]. Importantly, a substantial proportion of patients continue to experience moderate to severe pain despite low disease activity or clinical remission, suggesting that centralized pain mechanisms beyond peripheral inflammation play a crucial role in the persistence of chronic pain in RA [1,2,6].

Chronic inflammatory input from affected joints can induce peripheral sensitization and, over time, promote central sensitization (CS), resulting in amplified pain perception, allodynia, and hyperalgesia, which may persist independently of active inflammation [1,7]. These processes lower pain thresholds and enhance nociceptive signaling, contributing to chronic pain in RA even when synovial inflammation is clinically controlled [1,7]. Nociplastic-like pain features have been observed in a significant subset of RA patients. Screening studies utilizing tools such as the PainDETECT questionnaire have identified that approximately 15–25% of RA patients exhibit symptoms suggestive of neuropathic-like pain components [8]. While tools like PainDETECT are traditionally used for neuropathic pain screening, in RA, they often capture a broader ‘nociplastic’ phenotype, reflecting altered pain processing rather than overt structural nerve damage. Neuroimaging and quantitative sensory testing studies have demonstrated alterations in central pain processing pathways in RA, including widespread hyperalgesia and changes in pain thresholds, which are consistent with CS [9,10]. Proinflammatory cytokines, such as tumor necrosis factor-α (TNF-α), interleukin-1β (IL-1β), and interleukin-6 (IL-6), modulate nociceptive transmission by increasing peripheral nociceptor excitability and disrupting central pain inhibitory controls [1,7]. Notably, Janus kinase (JAK) inhibitors produce rapid analgesic effects, often preceding measurable reductions in traditional inflammatory markers. Preclinical and clinical evidence suggest that these agents may reduce both inflammatory and nociplastic-like pain by modulating cytokine signaling and neural sensitization pathways [11,12]. Taken together, these findings underscore the complexity of RA pain as a multidimensional phenomenon that arises from the interplay of inflammatory, neuropathic-like, and centrally mediated mechanisms, extending beyond synovial inflammation alone [7,11].

In this context, neuropathic pain—traditionally defined as pain resulting from a lesion or disease of the somatosensory nervous system—is characterized by burning, tingling, electric shock-like, or shooting sensations [13]. However, recent evidence suggests that a significant proportion of patients with rheumatoid arthritis (RA) experience nociplastic-like pain features, which arise from altered nociception in the absence of clear evidence of actual or threatened tissue damage that would typically activate peripheral nociceptors [8]. These pain mechanisms are clinically significant, as they are associated with higher pain intensity, increased fatigue, poorer sleep quality, mood disturbances, and significantly reduced quality of life [14,15]. Importantly, nociplastic-like pain features in RA may persist even with adequate control of synovial inflammation and often show limited responsiveness to conventional anti-inflammatory or disease-modifying therapies [13]. This highlights the clinical importance of recognizing non-inflammatory pain components in RA and supports the adoption of mechanism-based, multidimensional treatment strategies that target both inflammatory and centralized pain pathways to optimize patient outcomes [13,16].

Despite increasing recognition of neuropathic pain in rheumatoid arthritis (RA), the complex interrelationships among neuropathic pain features, inflammatory activity, and patient-reported outcomes remain incompletely understood [1,16]. While some studies have reported associations between systemic inflammatory markers and neuropathic pain [17], others emphasize the predominant role of central sensitization and non-inflammatory mechanisms in sustaining pain, even during periods of low disease activity or clinical remission [18]. Furthermore, prior research has frequently examined these factors in isolation or relied on limited assessment tools, resulting in an incomplete understanding of the multidimensional impact of neuropathic pain on patients’ daily functioning [18]. Consequently, a comprehensive approach integrating clinical, biochemical, and patient-reported measures—including fatigue, sleep quality, and health-related quality of life—is needed to clarify these complex interactions and to inform the development of targeted, mechanism-based interventions aimed at improving pain control and overall well-being in RA [17,18].

The aim of this study was to determine the prevalence and clinical characteristics of nociplastic-like pain features in patients with rheumatoid arthritis (RA) and to investigate their associations with pain intensity, disease activity, inflammatory markers, fatigue, sleep quality, and health-related quality of life. We hypothesized that nociplastic-like pain represents a substantial and distinct contributor to the overall disease burden in RA, occurring partially independently of systemic inflammatory activity, and that its presence is associated with significantly worse patient-reported outcomes. By integrating multidimensional assessments—including the PainDETECT questionnaire, Visual Analogue Scale (VAS), 36-Item Short Form Health Survey (SF-36), Functional Assessment of Chronic Illness Therapy–Fatigue (FACIT-F), and Pittsburgh Sleep Quality Index (PSQI)—this study aimed to provide a comprehensive characterization of nociplastic-like pain features in RA and support the development of more targeted, mechanism-based pain management strategies.

## 2. Materials and Methods

### 2.1. Study Design and Ethics

This cross-sectional study was conducted at the Faculty of Medicine, Dicle University, Diyarbakır, Türkiye. The study protocol was approved by the Dicle University Ethics Committee (Approval No: 2025-355), and all participants provided written informed consent in accordance with the principles of the Declaration of Helsinki. The primary objective of the study was to determine the prevalence and clinical characteristics of nociplastic-like pain features in patients with rheumatoid arthritis (RA) and to investigate their associations with disease activity, inflammatory markers, fatigue, sleep quality, and health-related quality of life.

### 2.2. Study Population

The study was planned and the literature review was initiated in January 2025. After obtaining approval from the Dicle University Ethics Committee, patient recruitment began. Between January and December 2025, 250 consecutive patients diagnosed with rheumatoid arthritis (RA) according to the 2010 American College of Rheumatology/European League Against Rheumatism (ACR/EULAR) classification criteria were screened at the Rheumatology Outpatient Clinic of Dicle University. Following a comprehensive clinical evaluation, 160 patients met the eligibility criteria and were included in the final analysis. The study was conducted in accordance with the Declaration of Helsinki, and written informed consent was obtained from all participants.

Inclusion criteria were: age ≥ 18 years, a confirmed diagnosis of RA, and sufficient physical and mental ability to complete clinical assessments and self-reported questionnaires.

To evaluate pain characteristics specifically within the context of RA and minimize confounding factors, strict exclusion criteria were applied. Patients were excluded if they had other autoimmune or inflammatory rheumatic diseases, severe neurological or psychiatric disorders, or comorbidities such as diabetes mellitus, peripheral neuropathy, chronic kidney or liver disease, or malignancy.

Additionally, patients using neuromodulatory drugs (e.g., gabapentinoids, antidepressants, or anticonvulsants) that could influence pain perception were excluded to avoid underestimating the true burden of nociplastic-like pain features. Comorbid fibromyalgia was specifically screened and excluded according to the 2011 Revised ACR Fibromyalgia Diagnostic Criteria [19]. These conditions were ruled out using a combination of patient self-reports and detailed review of electronic medical records, including rheumatology and neurology clinical notes, ensuring that no clinically overt peripheral neuropathy or secondary fibromyalgia was present in the study population.

Neuropathic-like (nociplastic-like) pain features were assessed using the PainDETECT questionnaire. Although originally developed for neuropathic pain, in the context of Rheumatoid Arthritis, this tool is increasingly recognized as a measure of the nociplastic phenotype, identifying sensory disturbances associated with central pain amplification. Consistent with contemporary rheumatology literature and the International Association for the Study of Pain (IASP) definition of nociplastic pain, PainDETECT scores were used to identify sensory qualities rather than to establish a definitive neurological diagnosis. Scores were categorized as unlikely (≤12), possible (13–18), or likely (≥19) nociplastic-like pain features. For binary analyses, patients with a score ≥ 19 were defined as the nociplastic-like pain (+) group, while those with scores ≤18 (including both unlikely and possible categories) were categorized as the nociplastic-like pain (−) group. This stringent cut-off was applied to maximize specificity and identify a distinct clinical phenotype.

Global pain intensity was measured using a 0–100 mm Visual Analog Scale (VAS). Disease activity was evaluated using the DAS28-ESR. Fatigue was assessed with the Functional Assessment of Chronic Illness Therapy–Fatigue (FACIT-F) scale, where higher scores reflect higher energy levels (less fatigue). Sleep quality was measured using the Pittsburgh Sleep Quality Index (PSQI), with higher scores indicating poorer sleep quality.

**Figure 1:**
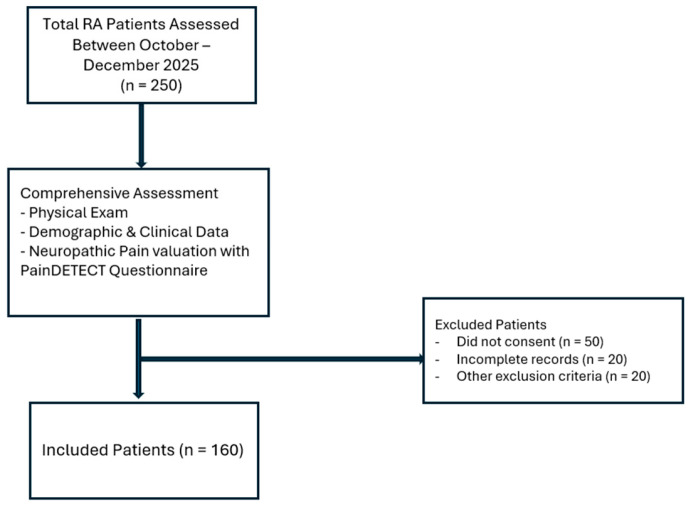
Study Population and Assessment for Rheumatoid Arthritis Patients.

All participants underwent a comprehensive clinical evaluation and completed standardized questionnaires under the supervision of trained researchers. The study population selection and assessment process are illustrated in Figure 1.

### 2.3. Clinical and Laboratory Assessment

Disease activity was assessed using the Disease Activity Score in 28 joints (DAS28), a composite index that incorporates tender joint count (TJC), swollen joint count (SJC), erythrocyte sedimentation rate (ESR), and the patient’s global assessment of disease activity measured on a 100 mm Visual Analogue Scale (VAS). Global pain intensity was evaluated separately using a 100 mm VAS, with higher scores indicating greater pain severity. To ensure biochemical accuracy, venous blood samples were collected after overnight fasting to measure C-reactive protein (CRP) and ESR using standardized laboratory methods. Demographic and clinical characteristics—including age, sex, education level, body mass index (BMI), and disease duration—were recorded at the time of assessment.

Given the focus on nociplastic-like pain features, particular attention was paid to the TJC as a potential indicator of centralized pain processing. Continuous variables were screened for normality, and those exhibiting a non-normal distribution—specifically CRP, ESR, disease duration, TJC, and SJC—were analyzed using non-parametric methods and are reported as median (interquartile range [IQR]). Additionally, education level was categorized to evaluate its potential role as an independent predictor of pain perception, as identified in the final multivariate stepwise regression model.

### 2.4. Patient-Reported Outcome Measures

Nociplastic-like pain symptoms were assessed using the PainDETECT questionnaire, a validated screening instrument designed to identify sensory qualities and symptoms related to central sensitization rather than to establish a definitive neurological diagnosis of neuropathic pain. The Turkish version of the PainDETECT questionnaire, adapted by Alkan et al., was used in this study [20]. Total scores range from −1 to 38. In this study, scores were categorized as unlikely (≤12), possible (13–18), or likely (≥19) nociplastic-like pain features. For binary analyses, patients were classified as “Likely nociplastic-like pain features (+)” (score ≥ 19) or “Negative for likely features (−)” (score ≤ 18). This stringent cut-off was applied to enhance the specificity of the nociplastic phenotype and minimize inclusion of ambiguous cases, consistent with previously validated protocols in RA research [21].

Fatigue was assessed using the Functional Assessment of Chronic Illness Therapy–Fatigue (FACIT-F) scale, which provides scores ranging from 0 to 52. Higher FACIT-F scores indicate greater energy levels, whereas lower scores reflect more severe fatigue [22]. Sleep quality over the past month was measured using the Pittsburgh Sleep Quality Index (PSQI), which yields a global score from 0 to 21; higher scores indicate poorer sleep quality, and a score > 5 is indicative of significant sleep disturbance [23].

Health-related quality of life was evaluated using the 36-Item Short Form Health Survey (SF-36), which assesses eight subscales spanning physical and mental health domains [24]. All questionnaires used in this study have been previously validated in Turkish. To ensure data integrity and consistency, all instruments were completed under the supervision of trained researchers during the outpatient clinic visit.

### 2.5. Statistical Analysis

All statistical analyses were conducted using IBM SPSS Statistics for Windows, version 27 (IBM Corp., Armonk, NY, USA). Continuous variables were assessed for normality using the Kolmogorov–Smirnov and Shapiro–Wilk tests, as well as visual inspection of histograms. Normally distributed variables are presented as mean ± standard deviation (SD), whereas non-normally distributed variables—specifically CRP, ESR, disease duration, and joint counts—are reported as median (interquartile range [IQR]). Categorical variables are expressed as frequencies and percentages.

Comparisons between patients with and without nociplastic-like pain features were performed using independent-samples *t*-tests for normally distributed data and the Mann–Whitney U test for non-normally distributed variables. Chi-square or Fisher’s exact tests were used for categorical variables. Effect sizes (Cohen’s d for *t*-tests and r for non-parametric tests) were calculated to quantify the magnitude of differences.

Correlations between PainDETECT scores and clinical or laboratory parameters were assessed using Spearman’s rank correlation coefficients (r_s_), given the non-normal distribution of key variables. Correlation strength was interpreted as very weak (r_s_ = 0.00–0.19), weak (0.20–0.39), moderate (0.40–0.59), strong (0.60–0.79), or very strong (0.80–1.0).

To identify factors independently associated with nociplastic-like pain features, stepwise multivariate linear regression analysis with bidirectional elimination was performed. This approach was used to minimize overfitting and identify the most relevant predictors from a candidate list including age, sex, education level, VAS pain, tender joint count, FACIT-F, and PSQI scores. Multicollinearity was assessed using variance inflation factors (VIFs), with VIF > 5 indicating potential collinearity. Model assumptions—including linearity, normality of residuals, and homoscedasticity—were verified using residual plots and the Durbin–Watson statistic.

All statistical tests were two-tailed, and *p*-values < 0.05 were considered statistically significant. A post hoc power analysis confirmed that the study was sufficiently powered to detect moderate effect sizes in the final cohort (*N* = 160).

## 3. Results

The final analysis included 160 patients diagnosed with rheumatoid arthritis (RA). The mean age of the study population was 51.41 ± 13.48 years, and the majority were female (n = 131, 81.8%). The mean body mass index (BMI) was 27.56 ± 5.20 kg/m^2^. Regarding educational attainment, 30.6% of participants were illiterate, 34.4% had completed primary education, 8.1% middle school, 16.9% high school, and 10% had a university degree. Most patients were married (76.9%), while 11.3% were single and 11.9% were widowed. The vast majority of the cohort were non-smokers (75.6%). Median disease duration was 8.0 years (interquartile range [IQR] 0.75–40.0). Assessment of nociplastic-like pain features using the PainDETECT questionnaire revealed a mean total score of 6.41 ± 2.45 for the entire cohort. Based on predefined cut-off scores, the distribution of pain phenotypes and their clinical correlates—including disease activity (DAS28), fatigue (FACIT-F), and sleep quality (PSQI)—are presented in the following sections.

Regarding pharmacological management, 31.2% of patients were receiving conventional synthetic disease-modifying antirheumatic drugs (csDMARDs). Among those on biologic or targeted synthetic therapies, 28.1% were treated with TNF inhibitors, 25% with JAK inhibitors, 7.5% with tocilizumab, 5% with abatacept, and 3.1% with rituximab. The mean DAS28 score was 2.73. Based on disease activity levels, 45% of patients were in clinical remission (DAS28 < 2.6), 20% had low disease activity (2.6–3.2), 28.1% had moderate disease activity (3.2–5.1), and 6.9% had high disease activity (>5.1). Analysis of pain phenotypes using PainDETECT scores revealed that 22.5% of patients were classified as having likely nociplastic-like pain features (score ≥ 19). Additionally, 15% were categorized as having possible nociplastic-like pain features (score 13–18), and 62.5% were unlikely to have such features (score ≤ 12) (Table 1).

Patients with likely nociplastic-like pain features demonstrated significantly higher pain intensity compared with those in the negative group (VAS: 60.89 ± 17.94 vs. 38.73 ± 18.58; *p* < 0.001). They also exhibited greater fatigue, as indicated by lower FACIT-F scores (23.13 ± 11.89 vs. 38.19 ± 12.61; *p* < 0.001), and poorer sleep quality (PSQI: 10.80 ± 3.35 vs. 7.08 ± 3.79; *p* < 0.001). While disease activity (DAS28: 2.87 ± 0.68 vs. 2.26 ± 0.86) and tender joint counts were significantly higher in the nociplastic-like pain group, no significant differences were observed in objective inflammatory markers, such as CRP and ESR, or in swollen joint counts. Health-related quality of life was markedly reduced in patients with nociplastic-like pain features, with significantly lower scores across all SF-36 domains, including physical functioning, role physical, role emotional, vitality, social functioning, mental health, bodily pain, and general health (all *p* < 0.001). Demographic factors, including age and BMI, did not differ significantly between groups, suggesting that nociplastic-like pain features may occur independently of systemic inflammation and basic demographic characteristics. Detailed comparisons of clinical, laboratory, and quality-of-life parameters are presented in Table 2.

The relationship between nociplastic-like pain features and sex was examined, revealing a statistically significant association (chi-square test, *p* < 0.001), with a higher prevalence of nociplastic-like pain observed among female patients. In contrast, no significant association was found between smoking status and nociplastic-like pain (*p* = 0.593). Furthermore, the type of antirheumatic treatment did not appear to influence pain phenotype; no statistically significant relationship was observed between treatment groups—including JAK inhibitors, TNF inhibitors, and conventional synthetic DMARDs—and nociplastic-like pain status (Pearson chi-square test, *p* = 0.707). These findings are detailed in Table 3, which presents the categorical distributions of pain phenotypes across demographic and treatment variables.

**Figure 2:**
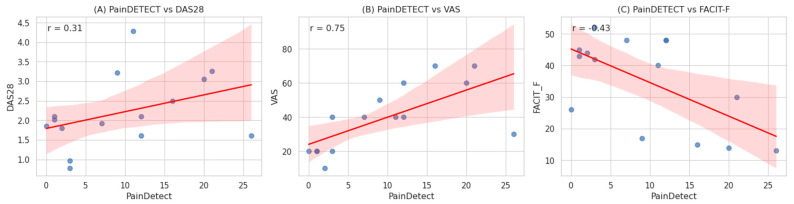
Correlations Between PainDETECT Scores and Clinical/Patient-Reported Outcome Measures in Rheumatoid Arthritis Patients. Scatterplots illustrating the relationships between PainDETECT scores and various clinical and patient-reported parameters in patients with rheumatoid arthritis. Each panel shows the Spearman correlation coefficient (r) between PainDETECT and the following variables: (**A**) Disease Activity Score in 28 joints (DAS28), (**B**) Visual Analogue Scale for pain intensity (VAS), (**C**) Functional Assessment of Chronic Illness Therapy–Fatigue (FACIT-F). Blue dots represent individual data points; the red line indicates the regression line with the shaded area showing the 95% confidence interval. All correlations were statistically significant (*p* < 0.01).

Spearman correlation analyses (Table 4, Figure 2) demonstrated significant associations between PainDETECT scores and various clinical and patient-reported outcome measures. PainDETECT scores showed a strong positive correlation with global pain intensity (VAS: r = 0.650, *p* < 0.01) and a moderate positive correlation with disease activity (DAS28: r = 0.527, *p* < 0.01). In contrast, weaker positive correlations were observed with objective inflammatory markers, including ESR (r = 0.235, *p* < 0.01) and CRP (r = 0.150, *p* < 0.01). Regarding patient-reported outcomes, fatigue (FACIT-F) was significantly negatively correlated with PainDETECT scores (r = −0.456, *p* < 0.01), indicating that higher nociplastic-like pain features are associated with greater fatigue severity (lower FACIT-F scores represent higher fatigue). Similarly, higher PainDETECT scores correlated with poorer sleep quality, as reflected by PSQI scores (r = 0.318, *p* < 0.01).

Health-related quality of life, as measured by SF-36 subscales, exhibited significant negative correlations with PainDETECT scores. The strongest associations were observed for Role Physical (r = −0.706), Social Functioning (r = −0.703), Physical Functioning (r = −0.690), Bodily Pain (r = −0.689), and Vitality (r = −0.634) (all *p* < 0.01). These findings indicate that nociplastic-like pain features substantially impair the physical, emotional, and social dimensions of quality of life in patients with rheumatoid arthritis.

A stepwise multivariate linear regression analysis with bidirectional elimination was performed to identify independent predictors of nociplastic-like pain features, as measured by PainDETECT scores. The final model was statistically significant (F = 72.8, *p* < 0.001) and explained 58.4% of the variance in PainDETECT scores (R^2^ = 0.584).

Among the candidate variables, pain intensity (VAS) (β = 0.352, *p* < 0.001) and tender joint count (TJC) (β = 0.138, *p* = 0.007) were identified as significant independent predictors, both showing positive associations with higher PainDETECT scores. In contrast, higher educational attainment was independently associated with lower PainDETECT scores (β = −0.115, *p* = 0.035), suggesting that education may influence the perception or reporting of nociplastic-like pain symptoms.

Variables including age, disease duration, inflammatory markers (CRP and ESR), and the composite DAS28-ESR score were excluded from the final model during the stepwise procedure, as they did not provide additional predictive value. Model diagnostics, including the Durbin-Watson statistic and variance inflation factors (all VIF < 2.0), confirmed an adequate model fit without evidence of multicollinearity or violations of regression assumptions (Table 5).

## 4. Discussion

The primary finding of this study is that nociplastic-like pain features in patients with rheumatoid arthritis (RA) are independently associated with perceived pain intensity (VAS), tender joint count (TJC), and education level, while showing no independent relationship with systemic inflammatory markers such as CRP and ESR [25]. Although global pain intensity emerged as the strongest predictor, the independent association with TJC—beyond its contribution to the composite DAS28 score—suggests that localized joint tenderness may, in part, reflect centralized pain processing rather than peripheral inflammation alone [26]. This dissociation highlights the significant role of central sensitization and non-inflammatory mechanisms in the RA pain experience, which may be underappreciated in routine clinical assessment [26]. Moreover, our multidimensional evaluation demonstrates that patients with nociplastic-like pain features bear a substantially higher disease burden, including increased fatigue, poorer sleep quality, and markedly impaired health-related quality of life [27]. These findings underscore the limitations of treatment strategies that focus solely on inflammation and emphasize the clinical importance of integrating approaches that target central pain mechanisms to optimize overall functional outcomes.

The prevalence of nociplastic-like pain features in our cohort (22.5%) is consistent with accumulating evidence indicating that approximately 20–40% of patients with RA experience such symptoms, even when systemic inflammation is clinically well-controlled [28]. Earlier studies using the PainDETECT questionnaire reported that 5–28% of patients were classified as having “likely” or “possible” nociplastic-like pain; however, more recent data suggest an even greater clinical burden [8]. A 2023 systematic review and meta-analysis corroborated these findings, reporting pooled prevalence estimates of 31–40% across various validated screening tools, including PainDETECT, DN4, and LANSS [28]. This upward trend is further supported by recent cross-sectional studies demonstrating that nearly half of RA patients exhibit clinical features of central sensitization [29]. Taken together, these findings—including those from the present study—reinforce the concept that persistent pain in RA is frequently driven by centralized nociplastic mechanisms, rather than being solely a reflection of peripheral joint inflammation.

Regarding demographic factors, our analysis revealed a significant female predominance in the prevalence of nociplastic-like pain features. This aligns with existing literature describing sex-based disparities in pain processing, where hormonal, neurobiological, and psychosocial factors likely contribute to lower pain thresholds and higher symptom burden among female RA patients [30,31]. Interestingly, while sex was a significant determinant, neither smoking status nor the specific class of antirheumatic therapy—including conventional synthetic DMARDs, TNF inhibitors, or JAK inhibitors—was significantly associated with nociplastic-like pain features [8].

The lack of correlation with treatment modality is particularly clinically relevant, suggesting that current biological and targeted synthetic therapies, although highly effective at suppressing peripheral inflammation, may not sufficiently modulate established central sensitization [32]. This observation reinforces emerging evidence that nociplastic-like pain in RA is often refractory to standard anti-inflammatory regimens. Consequently, achieving clinical remission based solely on inflammatory markers may not guarantee pain relief, highlighting the need for adjuvant strategies—such as neuromodulators or targeted non-pharmacological interventions—that address central pain mechanisms independently of disease-modifying therapies.

Our study found a moderate correlation between PainDETECT scores and DAS28 (r = 0.527). While this association suggests a link between disease activity and altered pain processing, it is crucial to interpret these findings with caution. The PainDETECT questionnaire, although effective in clinical settings, cannot definitively distinguish between structural neuropathic pain and nociplastic pain. In the context of RA, high scores likely reflect a nociplastic phenotype driven by persistent inflammation, rather than permanent neurological damage.

Although PainDETECT scores showed moderate correlations with inflammatory markers (CRP, ESR) and disease activity indices (DAS28), these associations were markedly weaker than the correlation observed with self-reported pain intensity (VAS). This discrepancy underscores that while inflammation contributes to pain in RA, it is not the sole driver of nociplastic-like pain features [33]. Our multivariate analysis further identified VAS pain intensity, tender joint count (TJC), and education level as the primary independent predictors of PainDETECT scores. The strong influence of VAS and TJC, independent of systemic inflammatory markers, supports the hypothesis that non-inflammatory, central mechanisms play a predominant role in these patients.

This interpretation aligns with recent evidence showing that nociplastic-like features can persist even in patients with well-controlled RA [34]. The coexistence of inflammatory and non-inflammatory inputs suggests a progression in which persistent peripheral nociceptive signaling leads to maladaptive neuroplastic changes. Over time, these changes promote central sensitization, amplifying pain responses that become increasingly resistant to conventional anti-inflammatory therapies [35]. Additionally, the independent association of lower education level with higher nociplastic symptom burden highlights the potential influence of psychosocial and socioeconomic factors on pain perception, emphasizing the need for a holistic, multidimensional approach to patient management.

Collectively, these findings confirm that nociplastic-like pain features constitute a distinct and clinically meaningful phenotype in RA, strongly associated with a heightened functional burden. Patients with this phenotype experience exacerbated fatigue, significant sleep disturbances (reflected by higher PSQI scores), and reduced health-related quality of life across all SF-36 domains [28]. Importantly, these impairments often persist despite low levels of systemic inflammation, highlighting the limitations of conventional, inflammation-focused pain management strategies.

Effective therapeutic approaches must therefore extend beyond the control of synovitis to specifically address central sensitization and nociplastic mechanisms [36]. Mechanism-based interventions may include neuromodulatory pharmacologic agents (e.g., gabapentinoids or SNRIs), cognitive-behavioral therapy, and structured physical activity programs [37,38]. Our results underscore the clinical utility of routinely assessing nociplastic features using validated instruments such as the PainDETECT questionnaire, enabling individualized, multimodal management for RA patients whose pain remains refractory to standard DMARD therapy and improving long-term functional outcomes.

From a clinical perspective, these findings highlight a potential “treatment trap.” Escalating immunosuppressive or biologic therapy in RA patients with high PainDETECT scores—particularly when inflammatory markers are low—may lead to overtreatment without achieving meaningful pain relief. Our data suggest that recognizing the nociplastic phenotype should instead prompt a shift toward mechanism-based interventions, including neuromodulatory agents such as gabapentinoids or SNRIs, as well as psychological and behavioral strategies aimed at modulating central pain processing.

The functional burden of nociplastic-like pain features in our cohort is substantial, characterized by significantly greater fatigue (FACIT-F), poorer sleep quality (higher PSQI scores), and lower health-related quality of life (SF-36), consistent with previous reports [8,28]. Strong negative correlations between PainDETECT scores and SF-36 domains—particularly Bodily Pain, Social Functioning, and Role Physical—underscore that nociplastic symptoms contribute meaningfully to overall disease burden, independent of objective measures of joint inflammation [28].

This burden is likely mediated by a self-perpetuating cycle in which chronic nociplastic pain enhances central sensitization, disrupting restorative sleep, exacerbating fatigue, and reducing psychological resilience [39,40]. Our finding that tender joint count (TJC) and education level—alongside global pain intensity (VAS)—serve as independent predictors of nociplastic-like features further clarifies this relationship. While VAS reflects the overall symptom burden, the independent contribution of TJC suggests a direct link to centralized pain amplification, and the influence of education level highlights the role of psychosocial resilience and health literacy in pain perception [32].

Although earlier studies often referred to “neuropathic pain” in RA, our findings align more closely with the IASP definition of nociplastic pain, characterized by altered nociception and central sensitization in the absence of identifiable nerve lesions. The features captured by PainDETECT in our cohort likely reflect these central processing alterations rather than classical peripheral neuropathy. Recognizing this distinction has critical clinical implications: persistent pain in RA may be refractory to standard anti-inflammatory escalation and instead requires mechanism-based, multidimensional management strategies. An integrated approach—including neuromodulatory pharmacotherapy, cognitive-behavioral interventions, and structured rehabilitation—is therefore essential for improving functional outcomes and overall well-being, rather than focusing solely on suppression of laboratory markers of disease activity [1,32].

Our study has several notable strengths. First, we evaluated a relatively large cohort of patients with RA, enabling a robust assessment of the prevalence of nociplastic-like pain features and their complex associations with disease activity and quality of life. A particular strength of our work lies in its comprehensive, multidimensional design: by integrating validated patient-reported outcome measures—including PainDETECT, SF-36, FACIT-F, and PSQI—with objective clinical and laboratory markers of inflammation, we were able to provide a nuanced characterization of the RA pain experience that extends beyond conventional joint counts.

Additionally, the use of stepwise multivariate linear regression allowed us to move beyond simple correlations and identify specific independent predictors—namely, pain intensity, tender joint count, and education level. This rigorous statistical approach offered critical insights into the non-inflammatory determinants of pain in RA, helping to distinguish nociplastic-like features from pain driven by peripheral synovitis. By employing standardized assessment tools within a well-defined cohort, our findings provide clinically meaningful data that can guide more personalized, mechanism-based strategies for pain management in rheumatology practice.

Despite these strengths, several limitations should be acknowledged. First, the cross-sectional design of this study precludes the establishment of causal relationships between nociplastic-like pain features, disease activity, and quality of life. It remains unclear whether nociplastic mechanisms amplify the perception of disease activity or if persistently high disease activity contributes to the development of these features. Second, although validated instruments were employed, reliance on patient-reported outcomes (PROs) introduces the potential for subjective perception and recall bias. Third, the absence of confirmatory diagnostic tools—such as quantitative sensory testing (QST) or formal neurological examinations—limits our ability to definitively classify pain types, and thus our findings should be interpreted as reflecting nociplastic-like features rather than formal neuropathic pain diagnoses. Fourth, the exclusion of patients receiving antidepressants or anticonvulsants, while necessary to avoid pharmacological confounding, may have led to the omission of individuals with more severe nociplastic-like symptoms. Consequently, the prevalence and severity reported here may underestimate the true spectrum of pain phenotypes in RA. Additionally, the exclusion of peripheral neuropathy and fibromyalgia was based on medical history and patient self-report rather than dedicated clinical screening, which may have allowed subclinical or undiagnosed cases to be overlooked. A notable limitation of this study is the use of the PainDETECT questionnaire, which is a screening tool rather than a diagnostic one. While it effectively identifies neuropathic-like sensory qualities, it cannot strictly distinguish between structural neuropathic pain and nociplastic pain. Therefore, our results should be interpreted as reflecting a ‘pain phenotype’ suggestive of centralized mechanisms. Finally, recruitment from a single tertiary care center may limit the generalizability of our findings to broader or primary care populations.

In light of these limitations, future longitudinal studies are warranted to rigorously investigate the temporal relationships between the development of nociplastic-like pain features and RA progression. Such studies should incorporate objective biomarkers, advanced imaging, or quantitative sensory testing to evaluate the long-term efficacy of targeted pharmacologic and non-pharmacologic interventions addressing central sensitization. Additionally, elucidating the neurobiological mechanisms underlying the transition from nociceptive to nociplastic pain will be critical for informing personalized therapeutic strategies. Ultimately, our findings highlight that effective RA management extends beyond the suppression of peripheral inflammation. By identifying pain intensity, tender joint count, and education level as independent predictors of nociplastic-like features, this study provides a foundation for refining clinical guidelines and implementing patient-centered, mechanism-based pain management. Integrating these approaches is essential for reducing the long-term burden of chronic pain and achieving holistic functional recovery in patients with RA.

## 5. Conclusions

This study contributes tothe current literature by providing a comprehensive, multidimensional assessment of potential nociplastic-like pain features in patients with rheumatoid arthritis. Our findings suggest that perceived pain intensity (VAS), tender joint count (TJC), and education level are associated with higher screening scores for nociplastic-like pain, highlighting the role of central pain processing mechanisms and psychosocial factors that may function independently of systemic inflammation.

We further observed that nociplastic-like pain features are linked to significantly greater fatigue, poorer sleep quality, and reduced health-related quality of life. These results underscore the clinical importance of routinely evaluating the suggestive nociplastic pain phenotype in RA, particularly when pain persists despite well-controlled inflammation. Collectively, our findings point toward the potential benefit ofpersonalized, mechanism-based management strategies—including neuromodulatory pharmacologic agents and behavioral interventions—that extend beyond conventional anti-inflammatory and disease-modifying therapies to improve holistic patient outcomes.

## Figures and Tables

**Table 1:** Demographic and Lifestyle Characteristics of RA Patients.

Variable		RA Patients (n = 160)
Age (years), mean ± SD		51.41 ± 13.48
Gender, n (%)	Female	131 (81.8%)
	Male	29 (18.2%)
BMI (kg/m^2^), mean ± SD		27.56 ± 5.20
Education Level, n (%)	Illiterate	49 (30.6%)
	Primary	55 (34.4%)
	Middle	13 (8.1%)
	High School	27 (16.9%)
	University	16 (10.0%)
Marital Status, n (%)	Single	18 (11.3%)
	Married	123 (76.9%)
	Widowed	19 (11.9%)
Smoking Status, n (%)	Non-smoker	121 (75.6%)
	Smoker	39 (24.4%)
Disease duration (years), median (IQR)		8.0 (0.75–40.0)
Prescribed RA medications, n (%)	csDMARDs	50 (31.2%)
	Anti TNFi	45 (28.1%)
	JAKi	40 (25%)
	Rituximab	5 (3.1%)
	Tocilizumab	12 (7.5%)
	Abatacept:	8 (5%)
DAS28 (0–10), mean ± SD		2.73 (0.77–6.35)
DAS28, n (%)	<2.6	72 (45%)
	2.6–3.2	32 (20%)
	3.2–5.1	45 (28.1%)
	>5.1	11 (6.9%)
Neuropathic Pain, n (%)	Likely nociplastic-like pain features	36 (22.5%)
	Possible nociplastic-like pain features	24 (15%)
	Unlikely nociplastic-like pain features	100 (62.5%)

RA, rheumatoid arthritis; SD, standard deviation; BMI, body mass index; IQR, interquartile range; n, number of patients; csDMARDs, conventional synthetic disease-modifying antirheumatic drugs; TNFi, tumor necrosis factor inhibitors; JAKi, Janus kinase inhibitors; DAS28, Disease Activity Score in 28 joints.

**Table 2:** Comparison of Clinical, Laboratory, Fatigue, Sleep Quality, and Health-Related Quality-of-Life Parameters Between RA Patients With and Without Nociplastic-like Pain Features.

Variable	Nociplastic-like Pain (+) (n = 36)			Nociplastic-like Pain (−) (n = 124)	*p* Value
Age (years), mean ± SD †	52.09 ± 13.64			51.19 ± 13.42	0.696
BMI (kg/m^2^), mean ± SD †	27.28 ± 6.31			27.76 ± 5.01	0.593
Education level, n (%) §			0.024		
Primary school or lower	24 (66.7%)	58 (46.8%)			
Middle school or higher	12 (33.3%)	66 (53.2%)			
Disease duration (years), median (IQR) ‡	7.0 (3.0–14.0)	8.0 (4.0–12.0)	0.340		
CRP (mg/L), median (IQR) ‡	6.2 (1.8–13.8)			4.4 (1.5–10.2)	0.341
ESR (mm/h), median (IQR) ‡	18.5 (10.0–33.5)			14.0 (7.0–21.0)	0.048
DAS28, median (IQR) ‡	2.9 (2.4–3.4)			2.3 (1.6–2.9)	<0.001
Tender joint count, median (IQR) ‡	1.0 (0.0–2.0)			2.0 (1.0–3.0)	0.038
Swollen joint count, median (IQR) ‡	0.0 (0.0–0.0)			0.0 (0.0–0.0)	0.906
VAS pain (0–100), mean ± SD †	60.89 ± 17.94			38.73 ± 18.58	<0.001
FACIT-F score, mean ± SD †	23.13 ± 11.89			38.19 ± 12.61	<0.001
PSQI score, mean ± SD †	10.80 ± 3.35			7.08 ± 3.79	<0.001
SF-36 Physical Functioning, mean ± SD †	57.56 ± 20.50			74.40 ± 19.07	<0.001
SF-36 Bodily Pain, mean ± SD †	34.56 ± 14.64			55.02 ± 20.14	<0.001
SF-36 General Health, mean ± SD †	22.78 ± 13.51			41.00 ± 18.76	<0.001

† Data are presented as mean ± standard deviation (SD); comparisons were performed using the independent samples *t*-test. ‡ Data are presented as median (interquartile range [IQR]); comparisons were performed using the Mann–Whitney U test. § Data are presented as number (percentage); comparisons were performed using the chi-square test. C-reactive protein (CRP), erythrocyte sedimentation rate (ESR), disease duration, and joint counts are presented as median (IQR) due to non-normal distributions, as determined by the Shapiro–Wilk test (*p* < 0.05). All other variables are expressed as mean ± SD. DAS28: Disease Activity Score in 28 joints; VAS: Visual Analogue Scale; FACIT-F: Functional Assessment of Chronic Illness Therapy–Fatigue; PSQI: Pittsburgh Sleep Quality Index; SF-36: Short Form-36 Health Survey.

**Table 3:** Distribution of Sex, Smoking, and Treatment Groups by Nociplastic-like Pain Status.

Variable	Nociplastic-like Pain (+) n: 36 (%)	Nociplastic-like Pain (−) n: 124 (%)	*p*-Value
Sex			<0.001
Female	29 (80.5%)	102 (82.2%)	
Male	7 (19.5%)	22 (17.8%)	
Smoking			0.593
Non-smoker	25 (69.4%)	96 (77.4%)	
Smoker	11 (30.6%)	28 (22.6%)	
Treatment Group			0.707
JAK	9 (25%)	31 (25%)	
TNF	11 (30.5%)	34 (27.4%)	
DMARD	12 (33.3%)	38 (30.6%)	
Others	4 (11.2%)	21 (17%)	

n, number of patients; %, percentage; *p*-value, significance value; JAK, Janus kinase inhibitors; TNF, tumor necrosis factor inhibitors; DMARD, disease-modifying antirheumatic drugs.

**Table 4:** Correlation Between PainDETECT and Clinical/Functional Measures.

Variable	r	*p*-Value
DAS28	0.527	<0.01
VAS (pain)	0.650	<0.01
ESR (mm/h)	0.235	<0.01
CRP (mg/L)	0.150	<0.01
FACIT-F (fatigue)	−0.456	<0.01
PSQI	0.318	<0.01
SF-36 PF	−0.690	<0.01
SF-36 RP	−0.706	<0.01
SF-36 RE	−0.567	<0.01
SF-36 VT	−0.634	<0.01
SF-36 SF	−0.703	<0.01
SF-36 MH	−0.665	<0.01
SF-36 BP	−0.689	<0.01
SF-36 GH	−0.661	<0.01

DAS28, Disease Activity Score in 28 joints; VAS, Visual Analogue Scale; ESR, erythrocyte sedimentation rate; CRP, C-reactive protein; FACIT-F, Functional Assessment of Chronic Illness Therapy–Fatigue; PSQI, Pittsburgh Sleep Quality Index; SF-36, 36-Item Short Form Health Survey; PF, Physical Functioning; RP, Role Physical; RE, Role Emotional; VT, Vitality; SF, Social Functioning; MH, Mental Health; BP, Bodily Pain; GH, General Health.

**Table 5:** Stepwise Multivariate Linear Regression Analysis of Predictors for PainDETECT Scores.

Step	Predictor Variable	β	Standard Error	t	*p* Value	95% CI for β
1	Constant	4.821	1.142	4.221	<0.001	[2.55, 7.08]
	VAS Pain	0.428	0.045	9.511	<0.001	[0.34, 0.52]
2	Constant	6.120	1.350	4.533	<0.001	[3.44, 8.80]
	VAS Pain	0.385	0.043	8.166	<0.001	[0.29, 0.48]
	Tender Joint Count	0.142	0.051	2.784	0.006	[0.04, 0.24]
3	Constant	7.450	1.520	4.901	<0.001	[4.43, 10.47]
	VAS Pain	0.352	0.044	8.000	<0.001	[0.26, 0.44]
	Tender Joint Count	0.138	0.050	2.760	0.007	[0.04, 0.23]
	Education Level	−0.115	0.054	−2.129	0.035	[−0.22, −0.01]

Final model explained 58.4% of the variance in PainDETECT scores (R^2^ = 0.584). Variables excluded by the stepwise procedure: age, disease duration, C-reactive protein (CRP), erythrocyte sedimentation rate (ESR), and DAS28-ESR. β: Standardized coefficient; t: t-statistic; CI: Confidence interval; VAS: Visual Analog Scale.

## Data Availability

The data supporting the findings of this study are available from the corresponding author upon reasonable request. Due to privacy and ethical considerations, these data are not publicly archived but can be provided to qualified researchers who meet the criteria for access to confidential information.

## Abbreviations

The following abbreviations are used in this manuscript:

SD	Standard Deviation
BMI	Body Mass Index
IQR	Interquartile Range
csDMARDs	Conventional Synthetic Disease-Modifying Antirheumatic Drugs
DMARD	Disease-Modifying Antirheumatic Drug
TNFi/TNF	Tumor Necrosis Factor Inhibitors
JAKi/JAK	Janus Kinase Inhibitors
DAS28	Disease Activity Score in 28 Joints
CRP	C-Reactive Protein
ESR	Erythrocyte Sedimentation Rate
VAS	Visual Analogue Scale
FACIT-F	Functional Assessment of Chronic Illness Therapy–Fatigue
PSQI	Pittsburgh Sleep Quality Index
SF-36	36-Item Short Form Health Survey
PF	Physical Functioning
RP	Role Physical
RE	Role Emotional
VT	Vitality
SF	Social Functioning
MH	Mental Health
BP	Bodily Pain
GH	General Health
